# Correlation between SD-OCT, immunocytochemistry and functional findings in an animal model of retinal degeneration

**DOI:** 10.3389/fnana.2014.00151

**Published:** 2014-12-22

**Authors:** Nicolás Cuenca, Laura Fernández-Sánchez, Yves Sauvé, Francisco J. Segura, Gema Martínez-Navarrete, José Manuel Tamarit, Lorena Fuentes-Broto, Ana Sanchez-Cano, Isabel Pinilla

**Affiliations:** ^1^Department of Physiology, Genetics and Microbiology, University of AlicanteAlicante, Spain; ^2^Departments of Ophthalmology and Physiology, University of AlbertaEdmonton, AB, Canada; ^3^Aragon Health Science Institute, IIS AragonZaragoza, Aragon, Spain; ^4^Department of Surgery, School of Medicine, University of ZaragozaZaragoza, Aragon, Spain; ^5^Bioengineering Institute, Miguel Hernandez University, ElcheAlicante, Spain; ^6^Bloss Group Company, Spain and Heidelberg Engineering GmbhHeidelberg, Germany; ^7^Department of Physiology, University of ZaragozaZaragoza, Aragon, Spain; ^8^Department of Applied Physics, University of ZaragozaZaragoza, Aragon, Spain; ^9^Department of Ophthalmology, Lozano Blesa University HospitalZaragoza, Aragon, Spain

**Keywords:** retinal degeneration, retinitis pigmentosa, P23H rat, visual acuity, electroretinogram, optical coherence tomography, autofluorescence, immunohistochemistry

## Abstract

Purpose: The P23H rhodopsin mutation is an autosomal dominant cause of retinitis pigmentosa (RP). The degeneration can be tracked using different anatomical and functional methods. In our case, we evaluated the anatomical changes using Spectral-Domain Optical Coherence Tomography (SD-OCT) and correlated the findings with retinal thickness values determined by immunocytochemistry.Methods: Pigmented rats heterozygous for the P23H mutation, with ages between P18 and P180 were studied. Function was assessed by means of optomotor testing and ERGs. Retinal thicknesses measurements, autofluorescence and fluorescein angiography were performed using Spectralis OCT. Retinas were studied by means of immunohistochemistry. Results: Between P30 and P180, visual acuity decreased from 0.500 to 0.182 cycles per degree (cyc/deg) and contrast sensitivity decreased from 54.56 to 2.98 for a spatial frequency of 0.089 cyc/deg. Only cone-driven b-wave responses reached developmental maturity. Flicker fusions were also comparable at P29 (42 Hz). Double flash-isolated rod-driven responses were already affected at P29. Photopic responses revealed deterioration after P29.A reduction in retinal thicknesses and morphological modifications were seen in OCT sections. Statistically significant differences were found in all evaluated thicknesses. Autofluorescence was seen in P23H rats as sparse dots. Immunocytochemistry showed a progressive decrease in the outer nuclear layer (ONL), and morphological changes. Although anatomical thickness measures were significantly lower than OCT values, there was a very strong correlation between the values measured by both techniques.Conclusions: In pigmented P23H rats, a progressive deterioration occurs in both retinal function and anatomy. Anatomical changes can be effectively evaluated using SD-OCT and immunocytochemistry, with a good correlation between their values, thus making SD-OCT an important tool for research in retinal degeneration.

## Introduction

Mutations in the rhodopsin gene are a common cause of retinitis pigmentosa (RP),^1^ with P23H being one of the most common autosomal mutations of this gene (Dryja et al., 1990), and accounting for approximately one third of RP cases in the USA (Dryja et al., 2000). The effects of this dominant mutation have been examined, at both anatomical and electrophysiological level (Machida et al., 2000; Cuenca et al., 2004; Pinilla et al., 2005), using the P23H transgenic rat (Lewin et al., 1998). However, these studies have relied on animals bred with an albino background, which is likely to increase the complexity of the retinal degenerative process. The use of pigmented heterozygous P23H animals provides a more precise model to study RP-related retinal degeneration. Furthermore, its slower degeneration rate is more suitable for the development and evaluation of preventive therapeutic approaches. The pigmented background also makes it easier to use functional evaluation tests for visual features such as visual acuity and contrast sensitivity.

The introduction of new optical imaging techniques such as Optical coherence tomography (OCT), a non-invasive method used to examine the retina *in vivo*, changed not only clinical practice, but also research. The fast speed acquiring images of Spectral-Domain OCT (SD-OCT) avoids artifacts and reveals retinal structures with high definition, and 3D visualization in a non-invasive manner. Some animal models had been previously examined with time-domain OCT, but resolution problems were encountered with the use of this technique (Horio et al., 2001; Li et al., 2001). However, SD-OCT has been successfully used to distinguish the retinal layers, with a good correlation with retinal histology in some animal models of retinal degeneration (Fischer et al., 2009; Huber et al., 2009). Clinical devices such as Spectralis OCT (Heidelberg Engineering, Germany), with minor modifications in their acquisition software, have been successfully used to evaluate rodent retinas (Fischer et al., 2009; Huber et al., 2009). The possibility of tracking retinal changes allows retinal degeneration to be monitored over time and therapeutic interventions to be evaluated *in vivo* in the same animal, thus reducing the number of animals required. The system can also be used to evaluate fundus autofluorescence (FAF), a method commonly used in clinical practice to diagnose retinal degeneration, and for angiographic imaging of retinal and choroidal vessels using fluorescein or indocyanine green.

In this study, we used functional and structural tests to study an animal model of RP, the P23H pigmented rat, paying special attention to the usefulness of SD-OCT for the detection of retinal changes in thickness and other features. Furthermore, these results were compared with those provided by immunocytochemistry.

## Material and methods

### Animals

Male pigmented transgenic rats, heterozygous for the P23H rhodopsin mutation, were bred from a cross between transgenic homozygous P23H Line 1 and normal pigmented Long Evans (LE) rats. The visual performance of the animals was monitored by means of an optomotor test given on a monthly basis (*n* = 8) and full field ERG recordings at P18, P21, P29, P58, P90 and P180 (*n* = 4). SD-OCT was performed at P130. Four normal LE rats crossed with Sprague-Dawley (SD) were used as wild-type controls at age P29. Transgenic rats were obtained from Dr. M. LaVail (UCSF), and bred in a colony at the University of Utah and at the University of Zaragoza, Spain, and maintained under a 12-hour light/dark cycle (light cycle illumination varied from 7 to 30 lux, depending on the front-to-back position within the respective cages). SD rats were obtained from Harlan Laboratories (Barcelona, Spain) and LE rats from Charles River Laboratories (Barcelona, Spain). They were housed and handled with the authorization and supervision of the Institutional Animal Care and Use Committees at both Universities. All procedures were performed in accordance with the ARVO Statement for the Use of Animals in Ophthalmic and Vision Research.

### Visual acuity and contrast sensitivity evaluation

To assess visual parameters, 8 rats were measured at P30, P60, P90, P120, P150 and P180, whereas control wild-type rats were evaluated at P30. The evaluation was carried out using an OptoMotry© system (CerebralMechanics, Lethbridge, Alberta, Canada) (Prusky et al., 2004; Douglas et al., 2005). The device consists of four screens positioned around a square testing chamber. An unrestrained rat is placed on a platform in the center of the square. A virtual cylinder consisting of a sine wave grating is drawn in 3D coordinate space and rotates around the animal, while it is recorded with a video camera. The spatial frequency of the grating is maintained at the viewing position by recentering the cylinder on the rat head. The cylinder is rotated at a constant speed (12°/sec). If the grating is seen by the animal, it tracks the stimulus with reflexive head and neck movements. Spatial frequency thresholds can be measured by systematically increasing the spatial frequency of the grating at 100% contrast until animals no longer track it. This threshold is considered to be maximum visual acuity. A contrast sensitivity curve was generated by identifying the minimum contrast that generates tracking, over a range of spatial frequencies.

Correlation between visual acuity and age was assessed using Pearson’s coefficient. Differences in contrast sensitivity between ages were evaluated using a Mann Whitney U test.

### Electroretinogram recordings

Full field ERGs were recorded at P18, P21, P29, P58, P90 and P180, with four animals being studied at each time point. Four normal LE rats crossed with SD were used as wild-type controls at age P29. Following overnight dark adaptation, animals were prepared for recording under dim red light. After being anesthetized with a mixture of ketamine (150 mg/kg i.p.) and xylazine (10 mg/kg i.p.), the animals’ head was secured with a stereotaxic head holder and their body temperature was monitored with a rectal thermometer and maintained homoeothermic at 38°C. Pupils were dilated using equal parts of topical phenylephrine (2.5%) and tropicamide (1%). Topical anesthesia with 0.75% bupivacaine was used to prevent any corneal reflexes, and a drop of 0.9% saline was applied regularly to the cornea to prevent dehydration and to allow electrical contact with the recording electrode (gold wire loop). A 25-gauge platinum needle inserted under the scalp between the eyes served as the reference electrode. Amplification (at 1–1000 Hz bandpass, without notch filtering), stimulus presentation and data acquisition were provided by a UTAS-3000 system from LKC Technologies (Gaithersburg, MD).

#### Mixed b-wave

For quantification of standard dark-adapted b-waves (showing aggregate rod and cone pathway contributions), the stimulus consisted of 3–5 single flash presentations (standard 10-µs duration). For intensity responses, stimuli were presented at sixteen increasing intensities, varying from −3.7 to 2.86 log cds/m^2^ in luminance. The effects of rod bleaching, which reduces the b-wave amplitude upon successive flashes, was minimized by increasing inter-stimuli-intervals (ISI), as the stimulus luminance was increased, from 10 s at the lowest stimulus intensity (−3.6 log cds/m^2^) up to 2 min at the highest stimulus intensity (1.4 log cds/m^2^). The maximum b-wave amplitude was that obtained during the flash intensity series, regardless of the specific stimulus intensity. Criterion amplitudes were established at 20 µV for a- and b-waves. The amplitude of the b-wave was measured from the a-wave negative peak up to the b-wave positive apex, rather than to the peak of oscillations, which can exceed the b-wave apex (Nusinowitz et al., 2001).

#### Isolation of cone response using a double flash protocol

The double flash protocol was similar to that employed in previous studies (Pinilla et al., 2004). A conditioning flash was followed 1 s later by a probe flash. The role of the conditioning flash in this paradigm is to transiently saturate rods so that they are rendered unresponsive to the probe flash. The intensity of the conditioning flash for complete rod bleaching was set at 1.4 log cds/m^2^ for all tests. The response to a probe flash (1.4 log cds/m^2^) preceded by the conditioning flash was understood to reflect cone-driven activity. The rod-driven b-wave was obtained by subtracting the trace of the response elicited by the probe from that elicited by the conditioning flash alone. An average of 3–5 traces (set 2 min apart to assure full recovery of rod responsiveness) were usually sufficient to obtain clear responses.

#### Additional characterization of cone responses using photopic adaptation

After testing as described above, rats were light adapted for 20 min, with a background illumination of 30 cds/m^2^ to reach a stable photopic response level. Photopic intensity-responses ranged from −1.6 to 2.9 log cds/m^2^ (−1.6, −0.6, 0.4, 1.4, 2.4 and 2.9 log cds/m^2^). Flicker responses to white strobe flash presentations with an intensity of 1.36 log cds/m^2^ were recorded. Stimuli were presented at 3 Hz, and were then increased in 5 Hz increments from 5 Hz to 50 Hz. When flicker fusion was achieved (3 µV criterion amplitude), the lower frequencies were studied and 60 responses were averaged.

### Fundus imaging system

For comparison with histopathological findings, six eyes from three P23H pigmented rats at P130 and four control rats were studied using a Spectralis HRA-OCT device. Rats were anesthetized by i.p. injection of a mixture of xylazine (10 mg/kg) and ketamine (90 mg/kg), and their pupils were dilated with tropicamide (Tropicamida^®^, Alcon labs, Barcelona, Spain). In order to improve the image acquisition quality, the rats wore a PMMA afocal contact lens, purchased from Cantoor Nissel (Market Place Brackley, Northamptonshire, UK). Tear substitutive drops were continuously added to improve transparence (Systane^®^, Alcon Labs, Barcelona, Spain). For examination, the animal was anesthetized using the same anesthetic mixture used for ERG recording, and placed on a platform mounted in the chin rest of the imaging device, with the eye in front of the system, so that the optic nerve could be easily visualized. A heat mat was used to keep the animals warm during the acquisition process.

Combined confocal scanning laser ophthalmoscopy (cSLO) and SD-OCT imaging was performed using a commercially available device (Spectralis HRA OCT system, Spectralis Eye Explorer 5.6b, Heidelberg Engineering, Heidelberg Germany). To adapt the system to the rat eye, a commercially available +25D lens was fit to the system (Heidelberg lens, HE 50744), and the length of the reference pathway was manually adapted according to the manufacturer’s instructions. The cSLO system used was an HRA2, using solid source for 488 and 514 nm and an infrared diode laser for 785 and 815 wavelengths. The protocol was always centered on the optic nerve. To evaluate the different thicknesses, one researcher (IP) manually changed the segmentation lines after acquisition. Values were obtained for total retinal thickness, Outer Nuclear Layer (ONL) + Retinal Pigment Epithelium (RPE) thickness, ONL thickness and Inner Plexiform Layer (IPL) thickness. Peripheral areas were used to assess intraindividual differences between superior and inferior retina thickness and temporal and nasal values. The mean values of all square areas, except those surrounding the optic nerve, were compared to evaluate differences between groups using a t test. These mean values were provided by the protocol used for the examination (Spectralis posterior pole protocol) using the optic nerve as the fixation position.

From each animal, a total of 5 single thickness profiles of the posterior map analysis were separately measured: crossing the optic nerve (section 31/61), middle upper (section 45/61) and upper (section 61/61), and middle lower (section 15/61) and lower (section 1/61). Thickness in each profile was measured at positions every 0.5 mm and compared between groups.

To acquire FAF images, fluorescence was excited using laser diodes at 488 nm and recorded between 500 and 700 nm. Fluorescein angiography was performed injecting 0.1 ml of 10% sodium fluorescein intraperitoneally.

### Immunostaining

Animals were euthanized with CO_2_ at P21, P40, P58, P90, P120 and P180, and their eyes were then enucleated. The eyecups were fixed in 4% paraformaldehyde in 0.1 M PBS at pH 7.4 for 1 h, and then washed in 0.1 M PBS before being cryoprotected in 10% sucrose for 1 h, 20% sucrose for 1 h and 30% sucrose overnight at 4°C. The next day, they were embedded in OCT medium and 10-µm thick retinal sections were cut on a cryostat in a horizontal plane and mounted on glass slides. Sections were treated for immunostaining as previously described (Cuenca et al., 2004), using the primary antibodies shown in Table 1. Slides were then mounted in watermount (Vector Labs) and coverslipped for viewing by means of confocal microscopy (Leica TCS SP2 Confocal System). Pinholes measured 77 µm and the widths of optical sections were 0.9 µm. Final images were obtained from the projections of 4–7 single frames. To control for non-specific staining, some sections were stained omitting the primary antibody.

**Table 1:** **Primary antibodies**.

**Molecular marker (Initials)**	**Antibody**	**Source and catalog number**	**Working dilution**
Bassoon	Mouse monoclonal	Stressgen (VAM-PS003)	1:1000
Calbindin D-28K (CB)	Rabbit polyclonal	Swant (CB-38a)	1:500
Protein kinase C, α isoform	Rabbit polyclonal	Santa Cruz Biotechnology	1:100
(PKCα)		(sc-10800)	
Rhodopsin (Rho)	Rabbit polyclonal	Chemicon-Millipore, #AB9279	1:200
		(Temecula, CA, USA)	
Transducin, Gαc subunit (Gt)	Rabbit polyclonal	Cytosignal (PAB-00801-G)	1:200
Recoverin	Mouse monoclonal	J. F. McGinnis, University	1 :1000
		of Oklahoma (Oklahoma City,	
		OK, USA	

### Relationship between OCT and immunochemistry measurements

For comparing the OCT and immunochemistry findings, four control and six P23H rat sections were measured at P120 to correlate the thickness of the different layers. The measurements for the histology sections were taken in the central area, close to the optic nerve in 8 retinal sections. The OCT measurements were made in the central retina with the values obtained previously described. ImageJ software (National Institutes of Health, Bethesda, MD, USA) was used for the morphometric analysis of confocal images. Results were analyzed by GraphPad Prism (GraphPad Software, Inc. La Jolla, CA 92037 USA). After checking for normal distribution using the Kolmogorov Smirnov test, a two-tailed Student’s *t*-test was performed to compare the OCT with immunohistochemistry measurements. *P* values equal to or less than 0.01 were considered to be statistically significant.

## Results

### Visual acuity and contrast sensitivity evaluation

**Figure 1:**
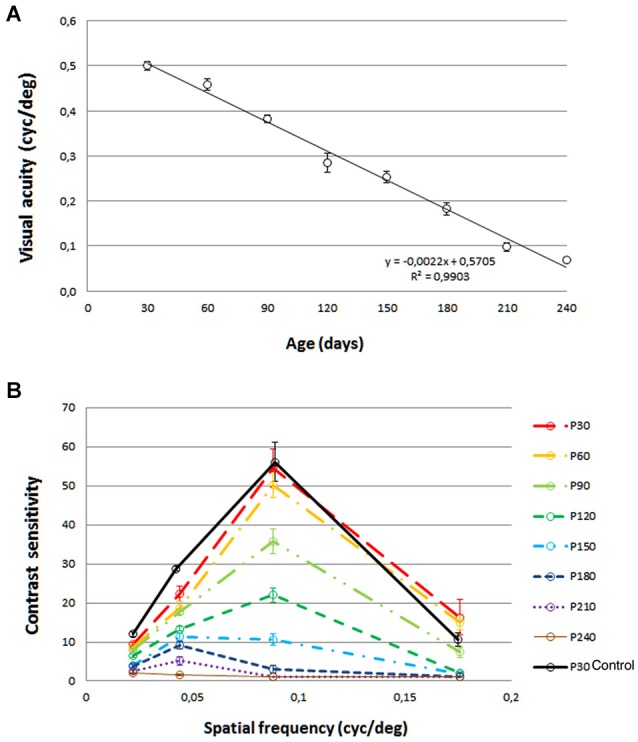
**Visual acuity (A) and contrast sensitivity (B) from rats aged 30 days to 240 days**. Values are compared to those of normal LExSD rats. Each point represents the mean value of 8 rats. Euthanization for histology limited the data at 150, 180, 210 and 240 days to 7 rats. Vertical bars represent the SEM. **(A)** Linear fit: Visual acuity = −0.0022 × age + 0.5705, R^2^ = 0.9903.

Visual acuity for control rats was 0.54 cyc/deg. A progressive visual acuity loss with age was observed in P23H (Figure 1A), with values of 0.500 cyc/deg at P30, 0.459 cyc/deg at P60, 0.382 cyc/deg at P90, 0.285 cyc/deg at P120, 0.253 cyc/deg at P150 and 0.182 cyc/deg at P180. This decline in visual acuity was well fit by a linear regression model, with a Pearson R^2^ value of 0.9903. The same trend was observed for the contrast sensitivity curve (Figure 1B). Peaks of 54.56 (P30), 50.30 (P60), 35.82 (P90), 22.04 (P120), 10.69 (P150) and 2.98 (P180) were obtained for a spatial frequency of 0.089 cycles/deg. The control rat value for this spatial frequency was 56.15.

Both the visual acuity and contrast sensitivity parameters began to decline at 30 days of age, and continued this trend throughout all the time points studied until the end of the experiment, when some degree of visual acuity still remained.

### ERG results

**Figure 2:**
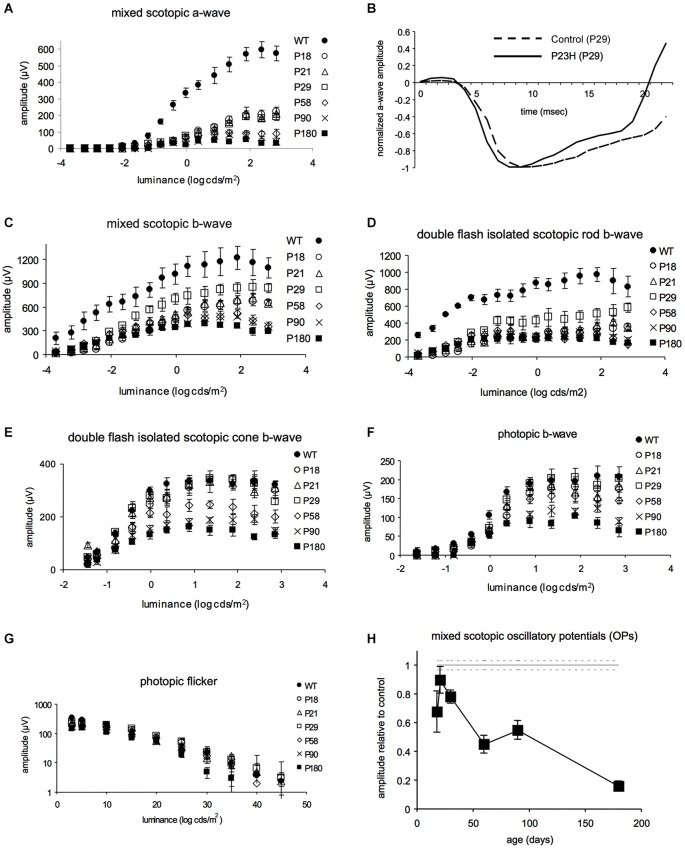
**Functional comparison by ERG between pigmented P23H L-1 and control rats. (A)** ERG amplitude *vs.* flash intensity (V-log I) series of mixed scotopic a-waves from LExSD and P23H rats. **(B)** Example of a-waves recorded at age P29 in normal (dotted line) and P23H (continuous line) rats. Amplitude values are normalized with respect to the maximal amplitudes; absolute amplitudes were 540 µV and 159 µV in normal rats and P23H rats, respectively. Stimulus luminance: 0.38 log cds/m^2^. **(C, D)** ERG amplitude vs. flash intensity (V-log I) series of mixed scotopic b-waves (panel **C**) and double flash isolated scotopic rod b-waves (panel **D**) Amplitudes increased from P18 to P29 in P23H rats and subsequently decreased in P23H rats with increasing age. **(E, F)** ERG amplitude vs. flash intensity (V-log I) series of cone b-waves recorded under scotopic (panel **E**) and photopic (panel **F**) adaptation. In P23H rats, amplitudes reached LExSD values from P18 to P29, and then progressively declined. **(G)** Amplitude of the flicker ERG response as a function of stimulus frequency for LExSD rats and P23H rats. Flicker amplitudes and fusions reached LE values at P29, and then dropped very slowly until P180 **(H)** Mixed scotopic OPs Values are expressed as percentage of P23H OP amplitude values relative to the average values in LExSD rats. The percentage increased from P18 to P29 and then gradually decreased until P180, when OPs were still clearly recordable. Error bars represent the standard error of the mean.

Scotopic a-waves were already reduced in amplitude at P18, achieving maximal values of 223 ± 27 µV (Figure 2A). Instead of increasing from P18 to P29, as would be expected (Dembinska et al., 2002) a-wave amplitudes reached a maximum of 206 ± 11 µV at age P29. This value amounted to only 34% of the corresponding value in wild-type LExSD rats aged P29 (604 ± 45 µV). Despite being affected at early time points, a-waves from pigmented P23H rats could still be elicited up to P180, the latest time points studied (Figure 2A).

In addition to being reduced in amplitude, the mixed scotopic a-waves in P23H rats had faster leading edges than in wild-type LExSD rats, indicating greater sensitivity. Figure 2B shows an example at 29 days of age, when the a-wave amplitude in P23H rats was only 29% of that in wild-type rats. Here, a-wave traces have been normalized against their respective maximal a-wave amplitude. As such, the resulting b-wave in P23H rats appears relatively higher than in wild-type rats because in the former the b-wave amplitude is relatively better preserved than the a-wave (see the trend of the respective ascending b-wave traces on the far right of Figure 2B).

At all ages, b-waves were less affected by the P23H rhodopsin transgene than were a-waves. While maximal b-wave amplitudes in both rod- and cone-driven ERG responses increased from P18 to P29, only cone-driven b-wave responses reached developmental maturity in pigmented P23H rats (Figures 2C–F). Although double flash-isolated rod-driven responses were already affected at P29, with a- and b-waves peaking at 203 and 564 µV vs. 610 and 998 µV in wild-type rats, these responses were still elicited at P180 (a- and b-wave maximal amplitudes of 60 and 211 µV). The rod-driven contribution to mixed scotopic b-waves was slightly lower in P23H (70%) as compared to LExSD (79%) rats, but remained steady at 58% by P180 (Figure 2D). Double-flash isolated scotopic cone b-waves (Figure 2E) and photopic single flash b-waves (Figure 2F) were comparable to those recorded in wild-type LExSD rats at P29. After P29, cone-driven single flash responses underwent slow, protracted deterioration (Figure 2F). By P180, maximal photopic b-waves still reached half of the amplitudes recorded in wild-type rats. Flicker ERGs (Figure 2G) confirmed the findings obtained with single flash cone-driven responses. Flicker amplitudes were comparable between P23H and wild-type rats at P29 (271 ± 30 µV *vs.* 306 ± 22 µV, respectively). Flicker fusions were also comparable at this age (42 ± 6 Hz and 42 ± 3 Hz in P23H and wild-type LExSD rats, respectively). By P180, flicker fusions in P23H rats were reduced in a statistically significant manner (31 ± 4 Hz compared to 42 ± 3 Hz in wild-type rats), as were maximal amplitude values (147 ± 19 µV in P23H rats *vs.* 306 ± 22 µV in control rats).

An indication of inner retina activity can be obtained by measuring the amplitude of oscillatory potentials (OP; Figure 2H). As with cone-driven b-waves, OP amplitudes increased from P18 until P29, at which point these values were comparable to those in age-matched wild-type rats. After P29, OP amplitudes decreased gradually to 16 ± 4% of the OP amplitudes in wild-type rats.

In summary, in P23H rats, only cone-driven b-waves (and mixed scotopic OPs) reached similar maximum amplitude values as in wild-type rats (Figure 2H). While mixed scotopic a-waves were the most severely affected ERG component, all components underwent a similar rapid reduction in maximal amplitude from P29 to P58, followed by a mild protracted drop from P90 to P180.

### OCT findings

**Figure 3:**
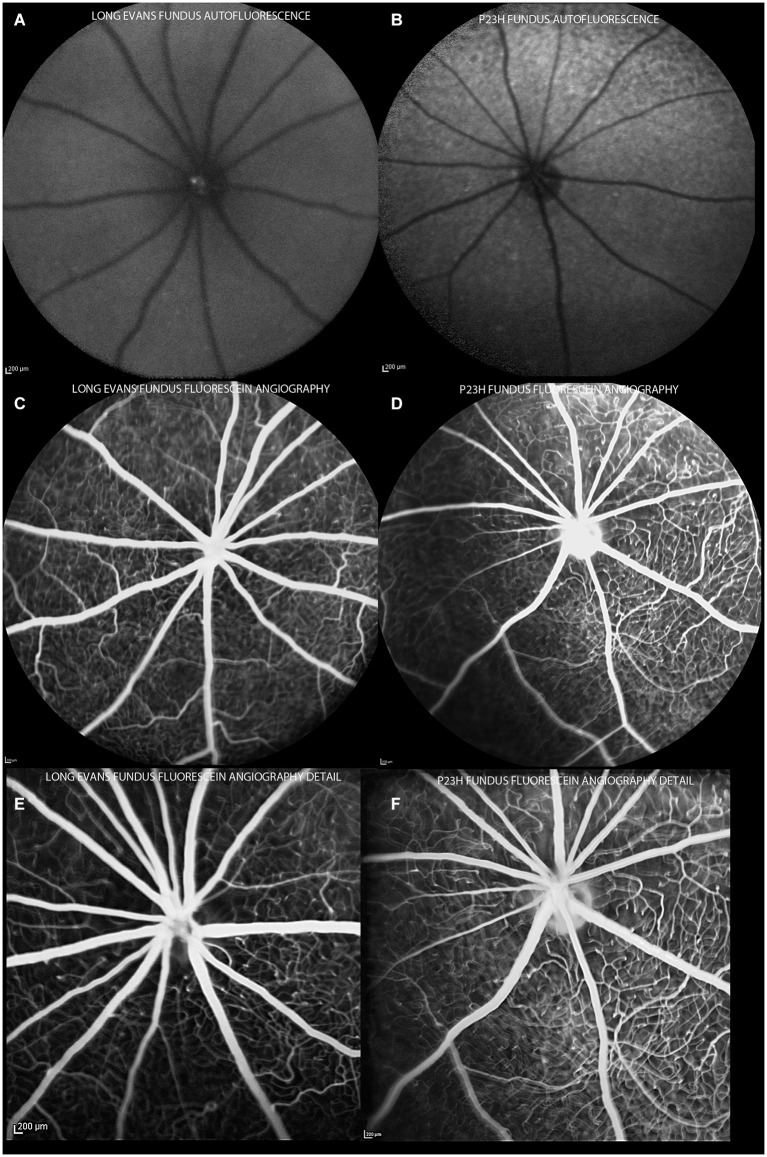
**Autofluorescence and angiography images of control and P23H pigmented rats at P130**. Autofluorescence images show a normal pattern in control rats **(A)** and hyperfluorescent spots in degenerative rats **(B)**. Angiograms of control **(C,E)** and P23H rats **(D,F)**, showing a normal radial pattern and capillary network at this age.

In all animals, a basic examination using en face cSLO imaging was performed to identify any retinal abnormalities. The examination included red-free (513 nm), infrared (830 nm) and FAF (492 nm) imaging. Fluorescence angiography was used to analyze retinal vessels (Figure 3).

**Figure 4:**
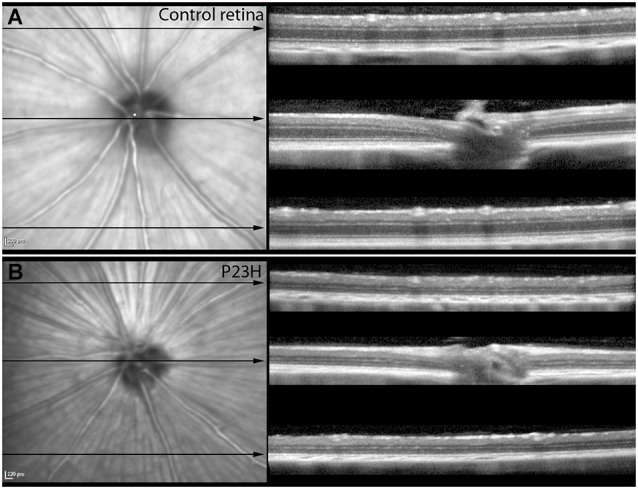
**Red-free images and OCT sections of control and P23H pigmented L1**. Red-free images and OCT sections of control rats **(A)** and P23H pigmented line 1 at P130 **(B)**. Red-free image showed the optic nerve and the major retinal vessels. The three sections showed the decreased thickness in the P23H rats, with a clear reduction of the ONL layer and changes in the hyperreflective layer. Note that the IPL is well preserved in the RP model.

Almost all the rats analyzed showed features of the Bergmeister papilla, a remnant of the developmental vascular vitreous. No other abnormalities were seen in the red-free and infrared examination (Figures 4A,B).

Control rats showed no autofluorescence in their fundus (Figure 3A). Change in FAF in the P23H rats was noted as sparse autofluorescent dots (Figure 3B). No relevant changes were observed in the angiography pattern (Figures 3C–F). Major retinal vessels emerged from the optic nerve in a radial fashion, and retinal vessels ramified and narrowed into a sparse capillary network. Both great vessels and the capillary network in P23H pigmented rat showed no permeability changes and no lost areas of capillary circuitry at this age.

**Figure 5:**
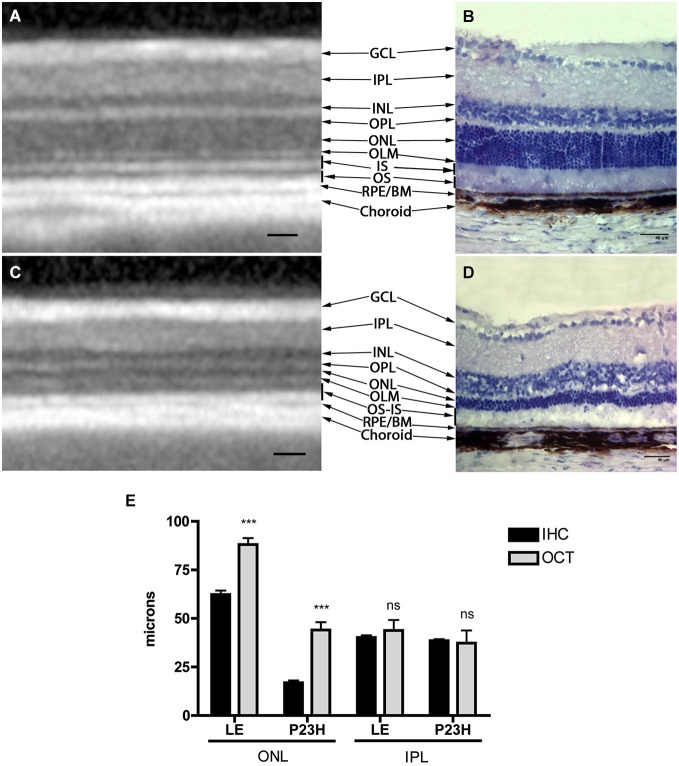
**Comparison between OCT and immunohistochemistry**. Figure shows an OCT section of a normal **(A)** and P23H rat retina **(C)** with hyper and hyporeflective layers and their correlation with normal **(B)** and RP **(D)** histology. The lower graph **(E)** shows retinal thickness values in healthy and diseased animals quantified by OCT and immunohistochemistry techniques. Mean values ± SD of different retinal layers measured by immunohistochemistry and OCT profiles. (***) *p* < 0.001. GCL: ganglion cell layer; IPL: inner plexiform layer; INL: inner nuclear layer; OPL: outer plexiform layer; ONL: outer nuclear layer; OLM: outer limiting membrane; IS: inner segments, OS: outer segments; RPE: retinal pigmented epithelium; BM: Bruch membrane. Scale bar: 40 µm.

**Figure 6:**
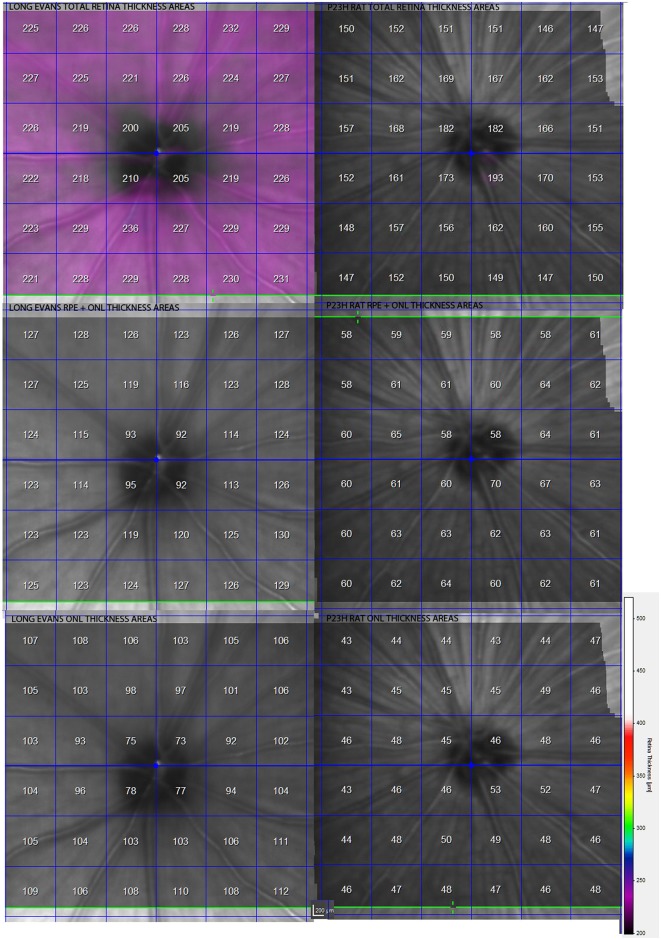
**Example of retinal maps of control and P23H pigmented line 1 rats at P130**. Mean retinal thickness value in microns is shown inside each square. Maps correspond to total retinal maps, RPE + ONL thickness and ONL thickness. All values were statistically lower in P23H rats.

Structurally, the sections showed changes between control and P23H rats. Control rats exhibited a normal OCT profile, with hyporeflectivity at the nuclear layers and hyperreflectivity in the plexiform layers and external retina, including outer segments, RPE and choroid. In normal rats, ganglion cells and their fibers are distributed together at the internal retina, limited by the internal limiting membrane (ILM); vessels were shown transversally cut with high reflectivity in their walls, distributed between the ganglion cells and the retinal nerve fiber layer. A hyperreflective IPL was followed by a hyporeflective INL, a high reflectivity OPL and a low reflectivity ONL. The four hyperreflectivity layers could be clearly observed at the external retina (Figures 4A, 5A; Spaide and Curcio, 2011). In transgenic rats, the ONL appeared clearly diminished in all the retinal profiles (Figures 4B, 5C). The RPE was disrupted and also diminished in thickness. Although the OPL was easy to find, the ELM showed gaps in its continuity and was more difficult to recognize (Figures 4B, 5C). The hyperreflective lines of the external retina were less evident in the P23H pigmented rat. The thickness of the IPL appeared almost normal. Figure 5 shows the retinal profiles obtained by OCT in control (Figure 5A) and P23H (Figure 5C) rats and its comparison with immunohistochemistry staining (Figures 5B,D). We applied both techniques in order to compare the data acquired in P23H and control rats at P120 (Figure 5E). The usefulness of OCT measurements for evaluating the progression of retinal disease is clear, albeit the data comparing their values with histology measurements are limited. Figure 6 shows an example of thickness maps of control and P23H rats. Mean peripheral areas were statistically higher in control vs. P23H rats. No differences were found between superior and inferior areas and nasal and temporal thicknesses in rats from the same group with any of the techniques.

All the values obtained in the retinal thickness profiles and maps of transgenic rats were significantly lower than those of control rats, with the exception of IPL thickness (Figure 5E). Total thickness retinal values were diminished in transgenic rats and the reduction was evident in the segmented layers (total retina thickness, ONL + RPE, and ONL). The main decrease was observed for ONL measurements. In addition, we found no differences between the data obtained with OCT and immunohistochemistry in the inner retina, while the outer retina showed differences between both techniques, with higher OCT (*p* < 0.001) than immunochemistry measurement values (Figure 5E).

### Anatomical findings

Anatomical studies were performed to compare them with the visual acuity, ERG and OCT image findings. For correlation with functional findings, we focused on cells involved in the generation of a- and b-waves, such as photoreceptors and their corresponding bipolar cells, respectively, and in the plexiform layers to check the synaptic relationship between the retinal cells.

#### Photoreceptors

**Figure 7:**
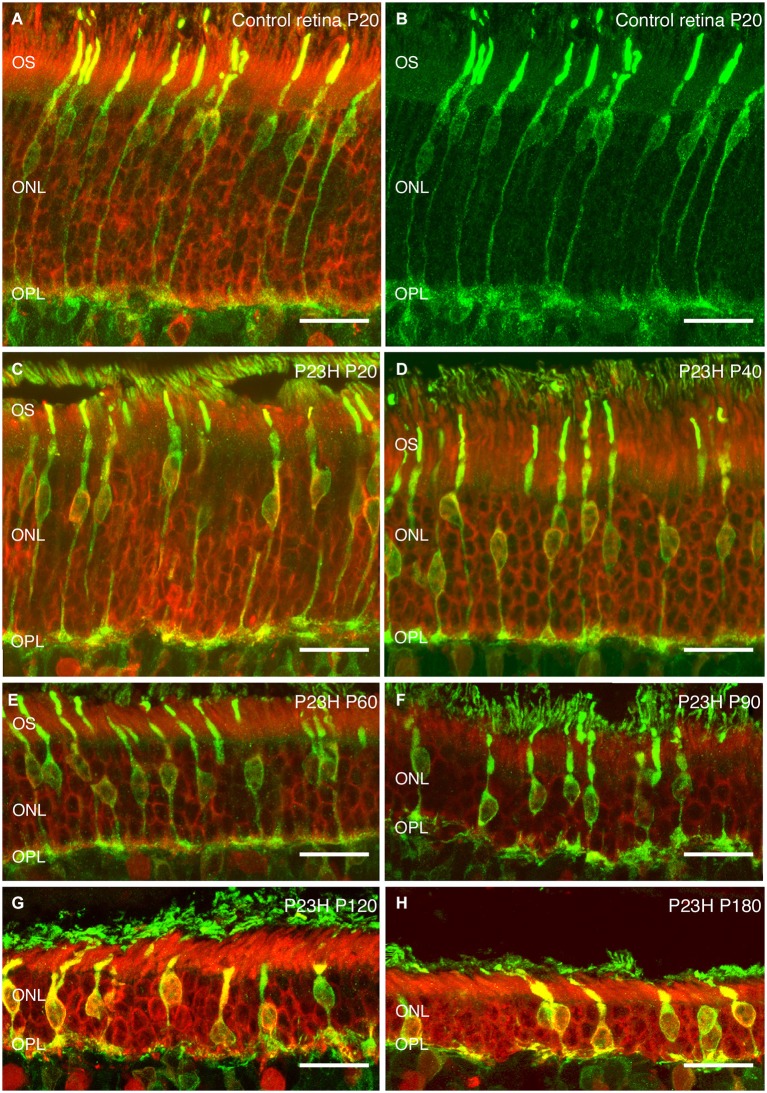
**Photoreceptor changes in P23H pigmented L1 compared to control rats**. Cryostat sections of a control (P20) and a P23H rat retina immunostained with antibodies against a specific marker for photoreceptor cells, recoverin (red); and a specific marker for cone and cone bipolar subtypes, γ-transducin (green). Photoreceptor cells in control rat retina stained with recoverin **(A)** and cone-photoreceptor cells stained with transducin **(A,B)**. P23H-L1 shows a decrease in photoreceptor rows and morphology changes from P20 to P180 **(C–H)**. OS: Outer segments; ONL: outer nuclear layer; OPL: Outer plexiform layer. Scale bar: 20 µm.

Recoverin was used to identify all photoreceptor cells, while cone photoreceptors were specifically stained using gamma-transducin (Figure 7). In wild-type rats, photoreceptor cell bodies were aligned in rows of up to 14–15 cells in thickness (Figure 7A). These cells displayed well-developed outer segments and axon terminals. Cone cell bodies were located in the external third of the ONL (Figures 7A,B).

At P20, the retinas of heterozygous P23H pigmented rats exhibited a similar number of photoreceptor rows as in wild-type rats. At this age, subtle changes in cone morphology were apparent: cell bodies were not merely confined to the external part of the ONL, rather they could also be found in the center of the ONL (Figure 7C).

At P40, the photoreceptor layer was reduced to 6–7 rows in thickness (Figure 7D). Cone cell bodies were distributed throughout almost the entire ONL. Cone outer and inner segments and axon terminals presented normal morphology, although the length of cone axon terminals looked shorter compared with control animals (Figure 7D).

These changes in length became clearer at P60, when the ONL was reduced to 4–5 cells in thickness and the outer segments showed the first signs of being truncated (Figure 7E).

The number of ONL cells was progressively reduced to 4–5 cells at P90 (Figure 7F) and 2–3 cell rows at P120 (Figure 7G). At both ages (P90, P120) (Figures 7F,G), the outer segments were clearly more truncated than at P60 (Figure 7E).

At the oldest age studied (P180), only 2 layers of photoreceptors remained in P23H retinas (Figure 7H). The proportion of cones *vs.* rods was increased, and the cones showed rounded cell bodies and increased immunoreactivity to transducin (Figure 7H). The overall cell length was severely reduced, most of the cones lost their outer segments and there was an obvious lack of axon and pedicles.

**Figure 8:**
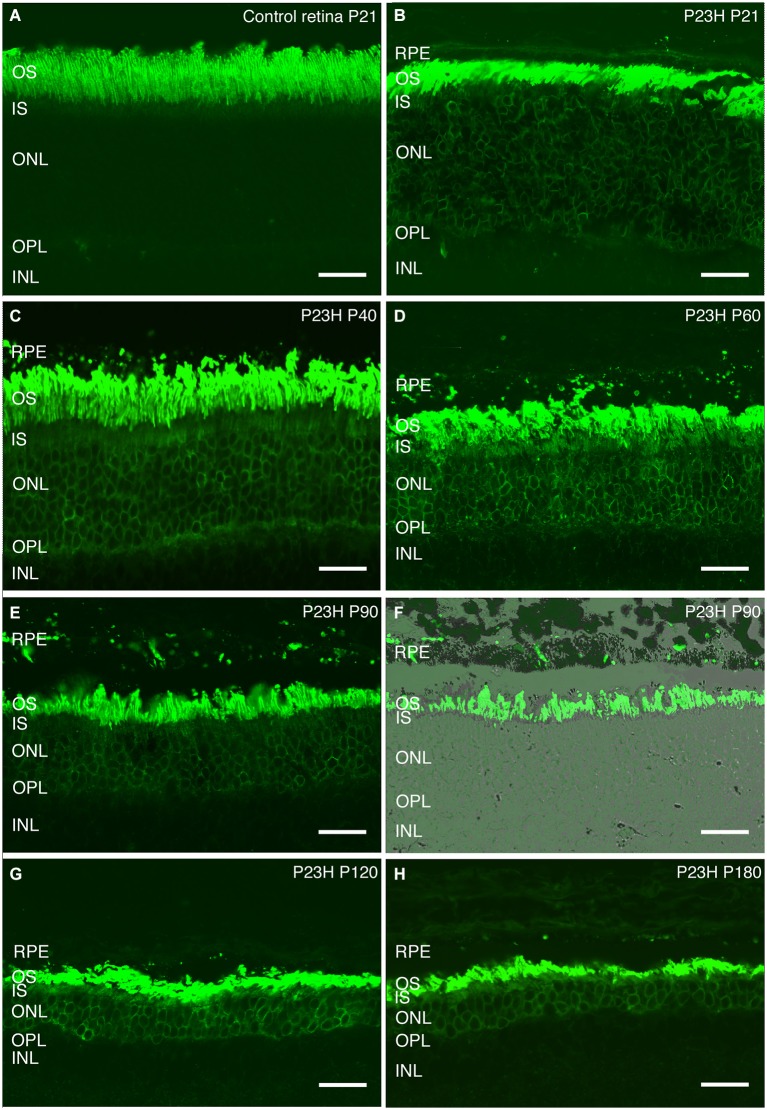
**Rhodopsin changes with the degeneration of P23H pigmented L1 rats**. Retinal sections stained with an antibody against rhodopsin showing rod outer segments in the control rat retina **(A)** compared to the P23H retina at the same age **(B)**. Ongoing retinal degeneration in P23H-L1 albino rats with aging **(C–H)** includes a progressive loss of photoreceptor rows, shortening of photoreceptor outer segments and mislocalization of rhodopsin along the cell. RPE: retinal pigment epithelium; OS: outer segments; IS: inner segments; ONL: outer nuclear layer; OPL: outer plexiform layer; INL: inner nuclear layer. Scale bar: 20 µm.

Rhodopsin is an essential protein involved in rod phototransduction, located specifically in the rod outer segment. Using antibodies against rhodopsin, the rods in LE rat retina (P21) showed a specific stain in their outer segments, with a well-organized and parallel distribution (Figure 8A).

Although the number of photoreceptors did not significantly decrease, rhodopsin staining in P23H pigmented rat retinas at P20 was no longer only localized in the outer segments (Figure 8B). Rhodopsin immunostaining could also be seen in the inner segments, the surface of rod cell bodies and their axons as a sign of rod degeneration at this age (Figure 8B). The mislocated rhodopsin staining of the rod inner segments and cell bodies was observed at all ages from P20 to P180 (Figures 8B–H). At P40, rod outer segments maintained their well aligned distribution (Figure 8C). However, at P60, they were shorter, truncated and had lost their parallel distribution (Figure 8D). An increase in rod outer segments phagosomes inside the RPE was observed at this age (Figure 8D). Figures 8E,F show phagosomes of rod outer segments inside the RPE at P90. The progressive impairment and shortening of rod outer segments could be observed at P120 and P180 (Figures 8G,F).

#### Synaptic connectivity changes in the OPL

The OPL is the layer in which synaptic contacts between photoreceptors and bipolar and horizontal cells take place (Kolb et al., 2001). We studied the morphological changes during the progression of photoreceptor degeneration at the OPL in the pigmented P23H rat. For this purpose, we used antibodies against calbindin to identify horizontal cells, PKC-α for visualizing ON-rod bipolar cells and bassoon to stain the synaptic ribbon on the axon terminals of cones and rods.

**Figure 9:**
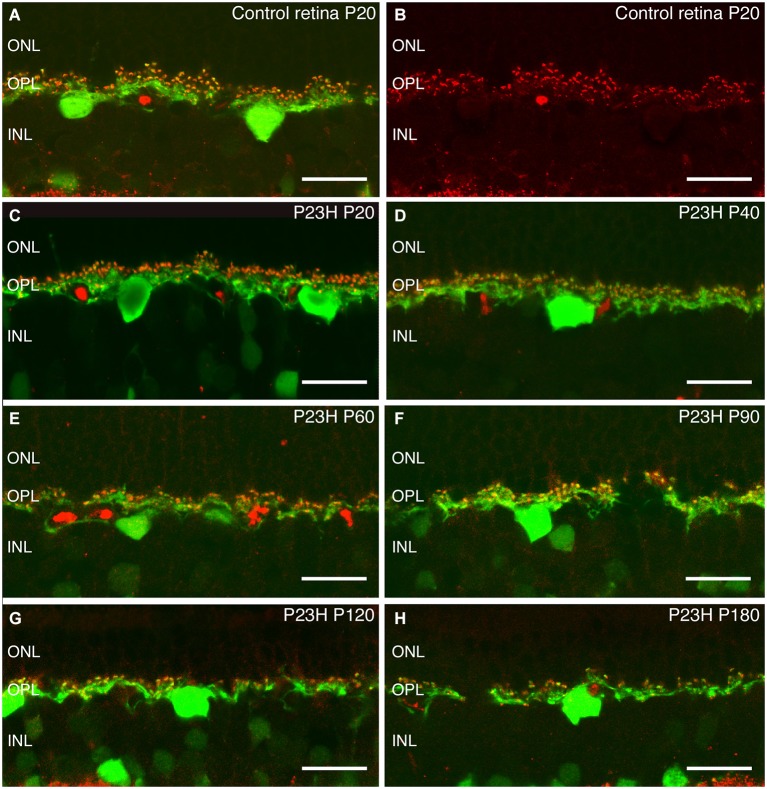
**Calbindin and bassoon staining of horizontal cells and synaptic ribbons in photoreceptor cells**. Immunostaining against calbindin (green), a specific marker for horizontal cells, and bassoon (red), a pre-synaptic ribbon marker. The staining shows the degeneration of synaptic contacts between photoreceptor and horizontal cells in P23H with age **(C–H)**, as compared to the normal rat retina **(A,B)**. ONL: outer nuclear layer; OPL: outer plexiform layer; INL: inner nuclear layer. Scale bar: 20 µm.

There is only one type of horizontal cell in the rat retina (Peichl and González-Soriano, 1994). It is a B-type horizontal cell with dendrites postsynaptic to cone pedicles and its axon making synaptic contacts with rod spherules. Figure 9 shows horizontal cell bodies and their axons immunostained with antibodies against calbindin (green). The synaptic ribbons of photoreceptors, identified with antibodies against bassoon (red), are located on top of horizontal tip terminals (Figures 9A,B red spots). As a result of rod photoreceptor degeneration in P23H retinas, the calbindin positive horizontal cells also showed degenerative changes (Figures 9C–H). At P20 and P40, the morphology and distribution of horizontal cells in P23H retinas were comparable to those of the control LE rat (Figures 9C,D), but bassoon immunoreactive spots were decreased, indicating a loss of contacts between rod and horizontal cells. By P60 and P90, there was a progressive disorganization of horizontal cell bodies, dendrites and axon terminals (Figures 9E,F, green processes). The cell bodies appeared to be in poor condition and were inverted, with atrophied dendrites condensing into blobs of stain. By 3 months of age, the horizontal cells in P23H retinas had sprouted abnormal processes from both dendrites and axon terminals trying to reach the remaining patches of photoreceptor synaptic terminals (Figure 9F). At P120 and P180, the horizontal cell processes appeared hypertrophic and beaded, and the number of process fibers were reduced and discontinuously distributed (Figures 9G,H). A loss of contacts with photoreceptor axon terminals was evidenced by the reduced number of bassoon immunoreactive spots (Figures 9G,H).

**Figure 10:**
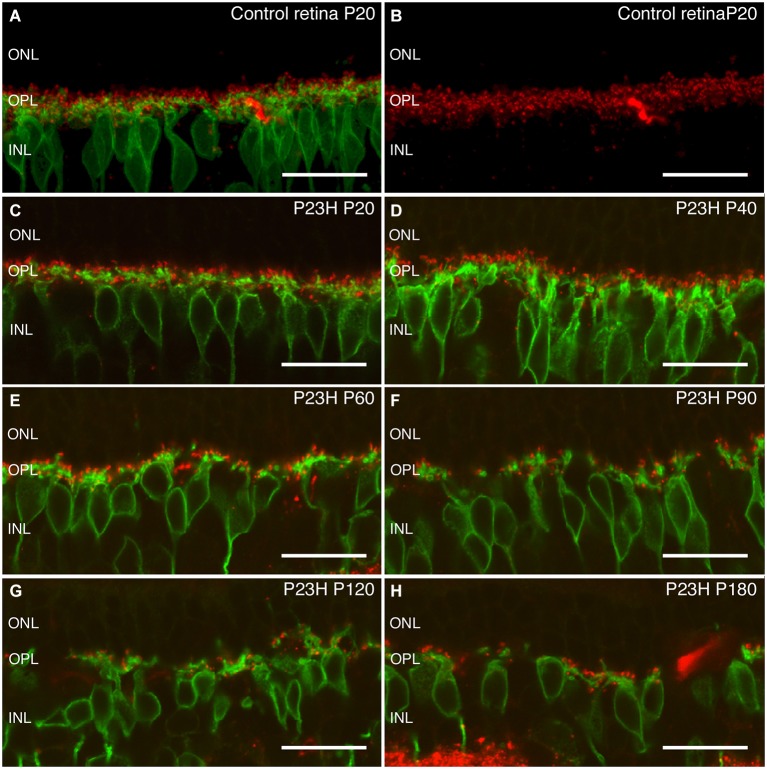
**Rod bipolar cells and ribbon synapses changes during retinal degeneration of P23H pigmented L1 rats**. Vertical sections stain with antibodies against protein kinase α (PKCα) in green to visualize rod bipolar cells, and against bassoon to visualize ribbon synapses in red. Control retinas show a dense plexus of synaptic contacts between bipolar and photoreceptor cells at the OPL level **(A,B)**. At P20, the P23H retina **(C)** already shows a small decrease as compared to control retinas. During aging **(C–H)**, there is a continuous reduction in the number of the synaptic contacts. ONL: outer nuclear layer; OPL: outer plexiform layer; INL: inner nuclear layer. Scale bar: 20 µm.

Rod bipolar cells are the only output cells of rod photoreceptors in the OPL. It is therefore important to see whether rod bipolar cells are particularly vulnerable during the course of rod photoreceptor degeneration. Rod bipolar cells can be labeled by antibodies against PKCα (Greferath et al., 1990). In control retinas, bipolar cells had a tuft of dendrites extending into the OPL, and double labeling with bassoon showed contacts with their presynaptic elements (Figures 10A,B). At P20 and P40, the morphology of rod bipolar cell dendrites in P23H rats was similar to that of the LE controls (Figure 10B, green), however a decrease of bassoon immunoreactive spots was evident (Figures 10C,D). At P60 and P90, the dendrites of rod bipolar cells in the OPL were thinner and shorter, and the OPL plexus was discontinuous and wavy, while a loss of contacts was also obvious Figures 10E,F. As degeneration proceeded, further changes became evident. Many of the cells lost their dendrites by P120 (Figure 10G, green) and a few bassoon immunoreactive spots could be observed (Figure 10G). By P180, a reduction of bipolar cells bodies took place. No dendritic plexus in the OPL was visible, and scarce bassoon spots were located directly on top of the cell bodies (Figures 10G,H), suggesting that the output of the remaining photoreceptors was dramatically impaired.

#### Synaptic connectivity changes at the IPL

To determine whether morphological changes took place in the IPL during photoreceptor degeneration, we studied two types of bipolar cells and the IPL bands stained with bassoon.

PKCα antibodies labeled ON-rod bipolar cells, and although some amacrine cells were also labeled, they can be easily distinguished by the characteristic distribution of their processes within the IPL.

##### Rod bipolar cells

**Figure 11:**
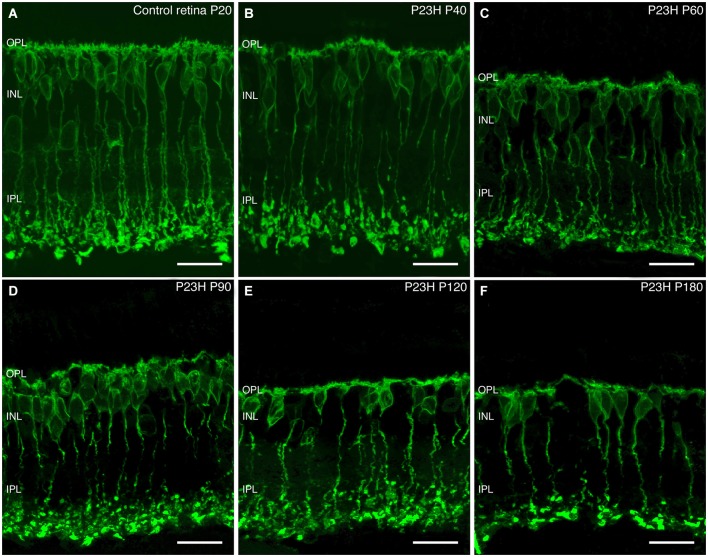
**Rod bipolar cell changes during retinal degeneration of P23H pigmented L1 rat**. Sections stained against PKCα for identifying rod bipolar cells in control retinas **(A)** and during P23H rat retinal degeneration **(B–F)**. Evident signs of rod bipolar cells degeneration started at P60; from this age and onward, there is a shortening of rod bipolar dendrites, a decrease in their immunoreactivity and loss of bipolar axon terminals. OPL: outer plexiform layer; INL: inner nuclear layer; IPL: inner plexiform layer. Scale bar: 20 µm.

In normal retinas, rod bipolar cell bodies were localized in the outer part of the INL (Figure 11A). These had a tuft of dendrites extending into the OPL and a single axon that extended through the IPL (with a few small side branches) and ended at stratum S5 of the IPL with end-bulbs and lateral terminal varicosities (Figure 11A).

ON-rod bipolar cells exhibited morphological changes during photoreceptor degeneration. From a normal morphology observed at P40 (Figure 11B), rod bipolar cells began to exhibit signs of degenerative changes at P60, with a reduction of their dendritic branches. Also, the cell bodies and axons lost their normal alignment and become shorter than in the control retinas (Figure 11C). Moreover, their axon terminal varicosities were smaller, and their axon terminal end clubs were particularly diminished. As the degeneration progressed from P60 to 180, rod bipolar cells experienced a reduction in number, had even fewer dendrites, and their axon terminal end-bulbs were also markedly less numerous (Figures 11C–F). Their axons became beaded and their terminal varicosities reduced. With further aging, the rod bipolar cell morphology became more disorganized, with cell bodies no longer displaying a regular laminar structure in the INL, and it was clear that the axon terminals were scarcely present at all (Figure 11F).

##### Cone bipolar cells

**Figure 12:**
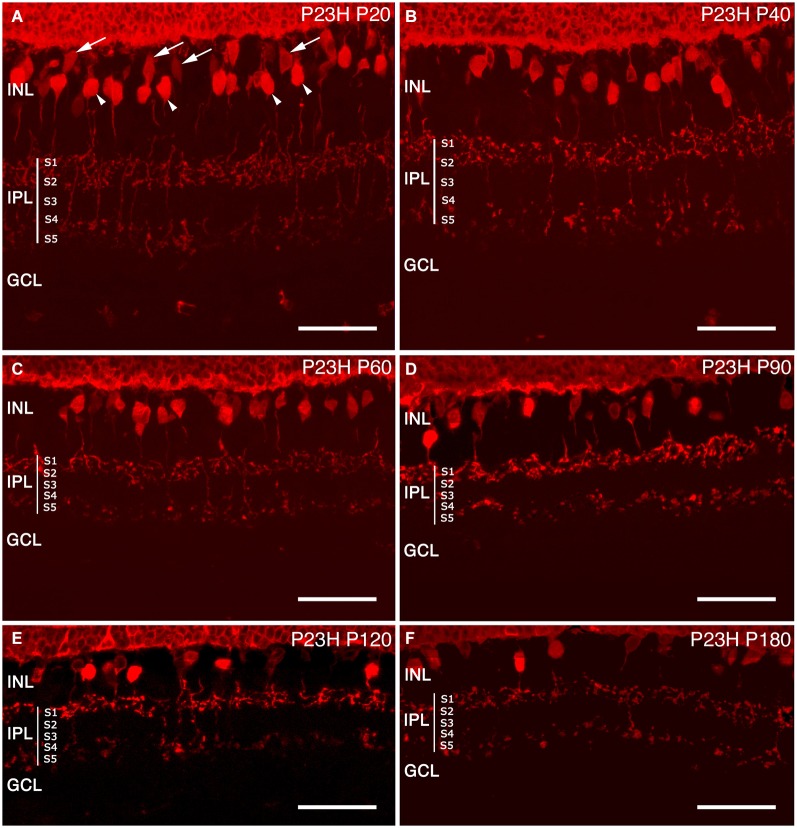
**Cone bipolar cell modifications during retinal degeneration of P23H pigmented L1 rats**. Retinal sections stained with antibodies against recoverin in order to visualize the degenerative process of cone bipolar cells in P23H **(B–F)** rat retina, compared to the control **(A)**. Along the degenerative process, cone bipolar cells show changes from P60 onwards, with profuse axonal loss at the IPL level. INL: inner nuclear layer; IPL: inner plexiform layer; S1: Stratum 1; S2: Stratum 2; S3: Stratum 3; S4: Stratum 4; S5: Stratum 5; GCL: ganglion cell layer. Scale bar: 40 µm.

In addition to photoreceptors, recoverin antibodies also label two types of cone bipolar cells in the rat retina (McGinnis et al., 1999; Cuenca et al., 2004). At P20, these two types of cone bipolar cells remained unchanged as compared to the control retinas. Type 8 ON-cone bipolar cells had a diffuse plexus of axons, terminating in the inner IPL, in strata S4-S5. Their cell body, located near the OPL, was weakly immunoreactive to recoverin (Figure 12A arrows). The second, Type 2, was an OFF-cone bipolar cell (Figure 12A, arrowheads), with strong immunoreactivity in the cell body, located near the middle of the INL, and an axon making a dense continuous plexus in strata S1 and S2 of the IPL. By P40 and P60, the general morphology of these cone bipolar cells was not drastically modified, and the only apparent change was a decrease in cell body density (Figures 12B,C).

At P90 and P120 (Figures 12D,E), the cell bodies of these two types of cone bipolar cells appeared smaller as compared to previous ages. The plexus in strata S1 and S2 was discontinuous, with gaps created by the disappearance of OFF-bipolar axon terminals. ON-bipolar cells were still present, but the terminals in stratum S5 appeared to be more sparsely distributed. At P180, the decrease of cone bipolar cells was evident and the axons of both cone bipolar cells were shorter, less complex and discontinuous, and their terminals exhibited a beaded morphology, indicating a loss of axon terminals (Figure 12F).

##### Bassoon at the IPL

**Figure 13:**
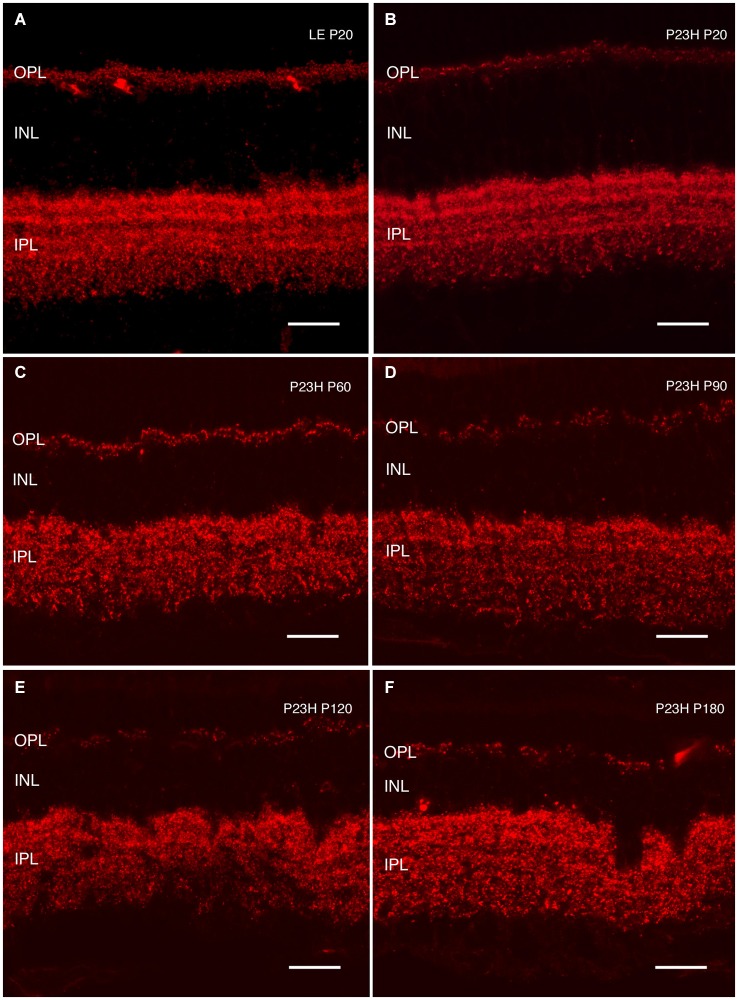
**Synaptic connectivity**. Cryostat sections stained against Bassoon (red) to visualize pre-synaptic ribbons. Bassoon immunostaining in P23H rat retinas was decreased **(B–F)**, as compared to control retinas **(A)**. A loss of connectivity was seen at the OPL, as well as increased disorganization at the IPL layer. ONL: outer nuclear layer; OPL: outer plexiform layer; INL: inner nuclear layer; IPL: inner plexiform layer. Scale bar: 20 µm.

In the IPL, bassoon is concentrated at the conventional GABAergic amacrine synapses, but is absent from the bipolar cell ribbon synapses (Brandstätter et al., 1999). Staining of vertical retinal sections of LE rats showed immunoreactivity in both synaptic layers of the retina (Figure 13A). Spots of bassoon immunoreactivity were found covering the entire IPL, with decreasing intensity from strata S1 to S5. There were distinct bands of strong immunoreactivity in strata S1, S2 and S3, with less intense staining at the S3/S4 border of the IPL (Figure 13A). At P20 (Figure 13B), bassoon immunoreactivity in the IPL remained unchanged in P23H rats as compared to LE rats. Bassoon staining was clearly diminished at P60, an age at which bassoon immunoreactive bands in the IPL were difficult to distinguish (Figure 13C). From P60 to P180, immunoreactivity decreased in synapses within the IPL over time, and the bands had become less distinct, in some cases disappearing altogether (Figures 13D–F).

## Discussion

The P23H rat is a dominant autosomal model of RP, used extensively for therapeutic approaches. The P23H albino rat model shows a faster retinal degeneration due to albinism, which make it quite different from the human disease. In contrast P23H pigmented rats present slowest degeneration being closer to P23H human condition. The retinas of genetically inbred albino rodents have been shown to be particularly susceptible to photic injury induced by moderate and high levels of light exposure (LaVail et al., 1987a,b; Naash et al., 1989). It is important to use a pigmented background, closer to the human retina, and to guarantee that pigmentation will not interfere with the retinal degenerative process. Pigmented rats are easier to use for visual acuity evaluation with the optomotor system and to define the lines of the OCT exploration. Their visual acuity resolution is higher than that of albino rats, which helps to distinguish the effect of the degeneration from the result of any therapeutic approach that we use on them. Not all rhodopsin mutations seem to have the same susceptibility to light damage. Some rodent models with faster retinal degeneration are more resistant to light exposure. For instance, retinal degeneration in the S334ter transgenic rat is not exacerbated by light exposure (Lowe et al., 2005). Other genetic factors have also been reported to be involved in the light sensitivity of different rodent strains (LaVail et al., 1987a,b,c). The heterozygous transgenic P23H albino rat has been found to be highly susceptible to retinal damage from exposure to intense light (Bicknell et al., 2002; Vaughan et al., 2003; Lowe et al., 2005). These studies point to the role of light damage in exacerbating RP-related degeneration in P23H mutant rats.

### SD-OCT as a non-invasive imaging technique to evaluate retinal degeneration

OCT is an important tool for retinal evaluation in all kinds of ophthalmological diseases. The fast acquisition and the high number of scans have made SD-OCT a valuable tool in ophthalmic research. The resolution of cross sectional images obtained by third generation OCT has been described as being similar to low power micrographs obtained from light microscopy (Huber et al., 2009). In our study, retinal thicknesses presented a good correlation with histological findings, and OCT proved able to assess the loss of retinal thickness with degeneration, as it showed a clear reduction in the ONL layer, as would be expected in a rod-loss disease. Other authors have shown a good correlation between retinal thickness measured by SD-OCT (Spectralis OCT) in wild-type mice and transgenic animals (rd1, Rho−/–, RPE65−/–) and histological findings (Huber et al., 2009; Jiao et al., 2013; Berger et al., 2014). Their results improved depending on the fixation protocol; thicknesses showed a better correlation when they used a direct embedding method as opposed to overnight fixation. Our previously described protocol used fixation and cryoprotection overnight, with increasing sucrose concentration. Using our standard protocol, the values obtained by immunohistochemistry are significantly lower than those measured *in vivo* during OCT exploration. However, the decreasing trend at the ONL level was clear with both techniques, and both methods exhibited an almost perfect correlation between them in terms of these measures. These findings were not supported by the IPL measurements, despite the fact that the IPL was the best preserved layer with both methods. The main reason for this finding is probably the difficulty in establishing a good limit for the layer, not only with OCT, but also histochemistry. The IPL is the latest layer to be affected, and its measurements remain close in value to those obtained in normal rats. In the immunohistochemistry examination, the S5 stratus was sometimes difficult to establish; OCT limits are clear at these ages, but it may be difficult to establish them in older rats (data not shown), due to cellular and vascular changes during retinal remodeling.

FAF findings are consistent with other retinal degenerative diseases, where photoreceptor debris and the accumulation of lipid degradation products are shown as hyperfluorescent dots. The findings resembled those found in RPE65−/– mice, but not so much as our own results for albino P23H rats (data not shown). Albino rodents are more susceptible to light-induced damage and show faster accumulation of lipid degradation products. P23H albino homozygous rats have an appearance resembling that of pigmented heterozygous rats at 2 months of age, with no changes at P21 (unpublished data). Albino wild-type mice BALB/c showed hyperfluorescent dots at early ages (Huber et al., 2009). Changes in FAF related to A2E accumulation has been described in other animal models of retinal degeneration (Charbel Issa et al., 2013).

Although is well known that retinal vessels change with degeneration (Villegas-Pérez et al., 1998; Fernández-Sánchez et al., 2012), Fluorescence angiography failed to show these changes at the age studied (4 months of age). All retinal vessels conserved their morphology, with no changes in their walls or any other signs or staining.

### Functional assessment using an optomotor test

The visual function of retinal degenerative animal models has been measured in previous studies using an OptoMotry system (Umino et al., 2006; McGill et al., 2007; Zhou et al., 2009; Lu et al., 2010). However, this study is the first to describe the spatial frequency and contrast sensitivity thresholds in the P23H-1 rat. Our results in control pigmented rats are similar to those reported by other studies (Douglas et al., 2005). Visual acuity reached 0.540 cyc/deg. The contrast sensitivity curve displayed a typical inverted-U shape. Both visual acuity and contrast sensitivity decreased with age in P23H-1 rats. At P30, visual performance was close to that found in control rats, and the results were consistent with those observed with ERG. As in control rats, the cone driven a-wave had reached maturity, but by this age, the rod driven b-wave was already reduced. Both functional tests demonstrate a gradual decrease in visual acuity, contrast sensitivity and ERG responses from the P30 age point to early in the P240 interval, showing a progressive photoreceptor deterioration that affects first the rods and then the cones. This agrees with the progressive loss of photoreceptors, which first affects the rods, and then both the rods and cones.

### Effect of albino vs. pigmented background in heterozygous P23H line 1 rats

The comparison of the present ERG results with previously published results from similar tests applied to heterozygous P23H line 1 rats, bred on an albino background (Machida et al., 2000), supports the hypothesis that albinism exacerbates the P23H-related retinal degeneration. While mixed scotopic maximal a-wave and b-wave amplitudes were initially better preserved in pigmented animals (at ages P29 and P28 in pigmented and albino rats, respectively), they underwent a similar decline up to the last time points tested (P180 and P203 in pigmented and albino rats, respectively). However, maximal photopic b-wave amplitudes did have higher proportions as compared to the respective wild types at early and late time points. These comparative findings support the suggestion that cone function might be more affected than rod function over time by the lack of pigmentation. Finally, the findings that the a-wave leading edge is faster in pigmented P23H than in wild types, as reported in albino P23H rats (Machida et al., 2000), suggests that pigmentation has no effect on rod phototransduction.

At the anatomical level, heterozygous P23H line 1 rats bred on an albino background showed a progressive loss of rod photoreceptors over several months. By P28, the ONL had lost approximately 40% of its cells (Machida et al., 2000). At this age, we observed rhodopsin mislocated along the remaining rod photoreceptor cells, which is a regular trait in RP diseases (Cuenca et al., 2004, 2005; Barhoum et al., 2008; Martinez-Navarrete et al., 2011). By P180, the ONL had fewer than two rows of cells remaining, and they were essentially cones. Our results indicate that pigmented P23H rats at age P21 had a similar number of photoreceptor rows as wild-type LE rats, while around 12 rows of cells remained at P40. This number then fell to 6 rows at P58, with a further progressive decline until P180, when only 2 layers of photoreceptors were left, consisting mainly of cone photoreceptor cells. As in other RP models reported by different group (Cuenca et al., 2014), the loss of rod photoreceptors leads to morphological changes in other retinal cells. Cone photoreceptor cells undergo changes affecting their length, the position of their cell bodies in the ONL, as well as changes in their outer segments, which become shorter and swollen than in control retinas.

The loss of photoreceptor cells is reflected as a reduction of synaptic contacts between photoreceptors and their postsynaptic bipolar and horizontal cells. Changes in bipolar cells were slower and more subtle than those reported in albino P23H rats (Cuenca et al., 2004): they became apparent only around P60, while this set of changes in bipolar cells appeared around P40 in the albino rat. The degenerative trends observed in the horizontal cell distribution and organization in P23H-L1 pigmented rats at P90 is almost achieved at P40 in the albino rat (Cuenca et al., 2004). All these observations confirm that the degenerative process that took place in the inner retina is closely related to the rod degeneration rate in each line, and should be considered in the design of RP therapies.

### Relevance to the development of preventive therapeutic approaches

The good correlation shown between OCT and histology results is the important key to our work. The possibility of evaluating the retina using non-invasive techniques reduces the number of animals that need to be sacrificed and still provides good data about the progression of the disease and the integrity of the retinal layers. Retinal thickness is not the only variable to provide valuable results; the autofluorescent findings also contribute information about the integrity of the RPE layer.

Pigmented RP rodent models have clear benefits over the albino ones. As in other models, albinism itself exacerbates the retinal degenerative processes. The changes seen in the inner retina are slower that those reported for the albino background in terms of the rate of decline in ERG responsiveness. Preservation of the retinal circuitry is an important factor in the effectiveness of potential preventive treatments. In addition, pigmented rats perform better on behavioral tests aimed at assessing vision. For these reasons, a pigmented background might be optimal when relying on P23H transgenic rats to develop and study experimental therapies, such as cell-based therapy.

The finding that cone function is unaffected by P29 provides us with the opportunity to study approaches aimed at the full functional prevention of blindness, using therapeutic approaches in the pigmented heterozygous P23H line 1 rat, providing that it is bred on a pigmented background. It should be noted that in our study, we were unable to detect a functional correlate of the subtle changes in cone cell body distribution at P21. Whether this morphological change has any impact on retinal function remains to be clarified.

## Author’s contributions

Experimental conceptualization and design: All authors

Experimental procedures and data interpretation: All authors

Data analysis: Isabel Pinilla, Yves Sauvé, Lorena Fuentes-Broto, Ana Sanchez-Cano, Nicolás Cuenca

Final discussion of the results: all authors

Drafting of the manuscript: Isabel Pinilla, Yves Sauvé, Laura

Fernandez-Sanchez, Ana Sanchez-Cano, Nicolás Cuenca

## Conflict of interest statement

The Associate Editor Alfonso Fairén declares that, despite being affiliated to the same institution as author Gema Martínez-Navarrete, the review process was handled objectively and no conflict of interest exists. José Tamarit works for Bloss-Heidelberg Company. Other authors declare that the research was conducted in the absence of any commercial or financial relationships that could be construed as a potential conflict of interest.

## Footnotes

^1^www.sph.uth.tmc.edu/RetNet/disease.htm
